# A deep cascaded segmentation of obstructive sleep apnea-relevant organs from sagittal spine MRI

**DOI:** 10.1007/s11548-021-02333-0

**Published:** 2021-03-26

**Authors:** Tatyana Ivanovska, Amro Daboul, Oleksandr Kalentev, Norbert Hosten, Reiner Biffar, Henry Völzke, Florentin Wörgötter

**Affiliations:** 1 Department of Computational Neuroscience, Georg-August-University, Friedrich-Hund Platz, 1, 37077 Göttingen, Germany; 2 Department of Prosthodontics, Gerodontology and Biomaterials, University Medicine Greifswald, Fleischmannstr. 42-44, 17475 Greifswald, Germany; 3 Institute for Physics, Alumni of University of Greifswald, Felix-Hausdorff-Str. 18, 17489 Greifswald, Germany; 4 Department of Radiology and Neuroradiology, University Medicine Greifswald, Fleischmannstr. 42-44, 17475 Greifswald, Germany; 5 Institute for Community Medicine, University Medicine Greifswald, Walther-Rathenau-Str. 48, 17489 Greifswald, Germany

**Keywords:** Sleep apnea, MRI, Segmentation, Deep learning

## Abstract

**Purpose:**

The main purpose of this work was to develop an efficient approach for segmentation of structures that are relevant for diagnosis and treatment of obstructive sleep apnea syndrome (OSAS), namely pharynx, tongue, and soft palate, from mid-sagittal magnetic resonance imaging (MR) data. This framework will be applied to big data acquired within an on-going epidemiological study from a general population.

**Methods:**

A deep cascaded framework for subsequent segmentation of pharynx, tongue, and soft palate is presented. The pharyngeal structure was segmented first, since the airway was clearly visible in the T1-weighted sequence. Thereafter, it was used as an anatomical landmark for tongue location. Finally, the soft palate region was extracted using segmented tongue and pharynx structures and used as input for a deep network. In each segmentation step, a UNet-like architecture was applied.

**Results:**

The result assessment was performed qualitatively by comparing the region boundaries obtained from the expert to the framework results and quantitatively using the standard Dice coefficient metric. Additionally, cross-validation was applied to ensure that the framework performance did not depend on the specific selection of the validation set. The average Dice coefficients on the test set were $0.89\pm 0.03$, $0.87\pm 0.02$, and $0.79\pm 0.08$ for tongue, pharynx, and soft palate tissues, respectively. The results were similar to other approaches and consistent with expert readings.

**Conclusion:**

Due to high speed and efficiency, the framework will be applied for big epidemiological data with thousands of participants acquired within the Study of Health in Pomerania as well as other epidemiological studies to provide information on the anatomical structures and aspects that constitute important risk factors to the OSAS development.

## Introduction

Obstructive sleep apnea syndrome (OSAS) is characterized by recurrent episodes of partial and complete airway obstructions during sleep with repetitive apneas and hypopneas [31]. This syndrome has one of the highest prevalence rates of sleep disorders in the general population, affecting approximately 3–7% and 2–5% of middle-aged male and female population, respectively [11], reducing significantly the patients’ life quality. Therefore, understanding of the causes and factors that influence this problem is of high importance. In our project, we were interested in the anatomical risk factors of OSAS. To achieve this goal, we aimed to extract and analyze such OSA-relevant structures as pharynx, tongue, and soft palate from numerous data acquired from the general population.

Whole-body magnetic resonance imaging (MRI) programs are usually utilized within epidemiological studies. Although a specially dedicated sequence, which depicts the organs listed above at high resolution, might not be available within the MR program, these organs are usually imaged. In our project, magnetic MRI data, which had been acquired with the Study of Health in Pomerania (SHIP) [18], were applied. SHIP is an on-going epidemiological study conducted in the northeastern Germany. Namely, sagittal T1- and T2-weighted sequences, that were primarily dedicated for spine imaging, appeared to contain the structures of interest for our project. Our goal was to develop a fast, fully automated, and efficient segmentation framework for pharynx, tongue, and soft palate structures.

In this paper, we present a cascaded framework for segmentation of the OSAS-relevant organs-of-interest from non-dedicated MRI sequences. Such a cascaded framework allows for efficient extraction of relatively small regions, which requires segmentation of unbalanced classes. The processing consists of several stages and is based on the well-known UNet architecture [4, 27]. First, the head and neck are extracted from the complete sequence, and the slices are cropped accordingly. Second, the pharyngeal structures are segmented slice-wise. Third, the extracted pharynx is utilized for the next cascade level. Basically, it is used for cropping of the tongue-relevant region, which the segmentation is applied to. Finally, the segmented tongue together with the pharynx defines the next cascade stage, and the central part of the soft palate structure is extracted there.

The paper is organized as follows. In Sect. 2, the overview of related works is given. We present and analyze the data used in this project in Sect. 3. Our method is thoroughly described in Sect. 4. The findings are presented in Sect. 5. Section 6 concludes the paper.

## Related work

Although computed tomography (CT) is the gold standard in otolaryngological routine [2], its application for research purposes on subjects without specific symptoms is not ethically justified. Cone Beam Computed Tomography (CBCT) requires a significantly lower radiation dose than a conventional CT, and it was also used for analysis of airways for sleep apnea patients [3, 8, 9, 21]. This imaging modality does not allow for analysis of soft structures, such as tongue and soft palate, though.

Since the ultimate goal of our project includes big data analysis of airways as well as neighboring soft structures, which are hardly distinguishable in CT and CBCT, only methods for MRI data are of interest.

There are several MRI-based approaches for analysis of the throat region described in the literature. Some studies, for instance, by O’donoghue et al. [24], investigated upper airway anatomy from MR scans using only manual delineation of organ boundaries. Such an approach is prone to inter- and intra-observer variability and requires a lot of human working hours. For analysis of data from hundreds of subjects, this approach is hardly applicable.

Abbot et al. [1] analyzed airway volumes from children with OSA using K-means clustering algorithm in a semiautomatic manner. Trushali et al. presented a pharynx and larynx cancer segmentation framework for automatic base of tongue and larynx cancer segmentation from T1-weighted magnetic resonance images (MRI) [7] in axial projection. The authors applied classical algorithms, such as fuzzy c-means and level sets [12] to extract the cancerous regions. Segmentation of airways was implemented as the first preprocessing step to define the region of interest. Campbell et al. proposed a 3D level set approach for pharynx segmentation from axial MR data [5]. However, the results were quantitatively evaluated only on synthetic data. Ivanovska et al. [15] proposed a minimally interactive method for pharynx segmentation from axial T1-weighted MR sequence using classical algorithms and knowledge about location of the pharyngeal structures. The approach was further extended to be a fully automated [16] one. Shahid et al. presented an algorithm consisting of visual feature space analysis for selection of a 3D pharyngeal structure and refinement with intensity-based methods [30] for automated pharynx segmentation on the similar dataset. These methods were proposed to segment only a small part of the structure in the retropalatal oropharyngeal region from the MR sequence with the 1 mm resolution in each dimension. These methods were based on the fact that slices in axial projection had a sufficient resolution to separate pharynx from the other close structures and artifacts.

In the last years, the deep learning approaches gained a tremendous popularity and proved their efficiency also in medical image analysis tasks [13, 29]. Tong et al. proposed a shape-constrained GAN-DenseNet for multi-organ segmentation from CT and low field MR data [32]. The average Dice coefficient for pharynx segmentation is 0.706. Erattakulangara and Lingala [10] proposed to apply U-Net to segment the vocal tract from mid-sagittal MRI images and achieved DICE coefficient of $90\%$.

There are few methods for segmentation of tongue structures. Peng et al. [26] proposed a variational framework to assess tongue contours from mid-sagittal images. However, no complete tongue boundaries were obtained there. Harandi et al. [20] tackled a 3D semiautomatic tongue segmentation with inter-subject mesh-to-image registration scheme and achieved DICE coefficient of $90.4\%$. Their dataset consisted of 18 subjects and the data resolution was $256\times 256\times z$ (*z* ranged from 10 to 24) with 0.94 mm $\times$ 0.94 mm in-plane resolution and 3 mm slice thickness.

Soft palate region was analyzed in several works. Chen et al. [6] extracted a computational three-dimensional (3D) soft palate model from a set of MRI data to generate a patient-specific model. The segmentation was performed manually. Ogawa et al. [25] assessed tongue and soft palate measurements in MR data of obstructive sleep apnea patients. The measurements were also performed manually. To our knowledge, there are no publications addressing automated segmentation of the soft palate region from mid-sagittal MRI.

## Materials

In our previous work [15, 16, 30], we utilized an isotropic T1-weighted head sequence from the SHIP database. However, this sequence does not contain some of the structures, such as oral cavity, which are of interest in the current project.

**Fig. 1:**
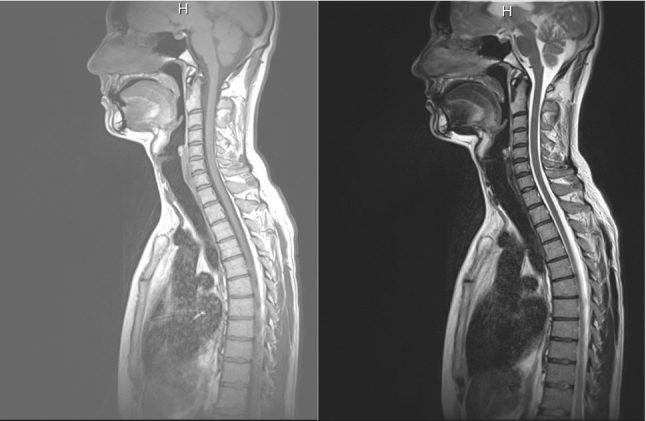
Example slices from T1-weighted (left) and T2 (right) sequences

Hence, a different sequence had to be chosen. Namely, T1-weighted and T2-Weighted TSE (turbo spin echo) sequences were utilized. The T1- and T2-weighted sequences were taken in one measurement round, i.e., registered by acquisition. These data in sagittal plane were primarily acquired for spine analysis and contained only the central part of the human body, which also included airway and soft structures of interest for our project. The resolution was $1.116\times 1.116 \times 4.4$ mm$^3$. The spatial resolution was $448\times 448$, and the number of slices varied from 15 to 19. Such a resolution after conversion to axial plane was not sufficient for successful application of previously developed methods [16, 30], since the slice width in the axial projection would be 15 to 19 pixels only. In Fig. 1, example slices from T1- and T2-weighted sequences are shown.

The experienced observer manually delineated several OSA-relevant structures, namely: pharynx, tongue, and the central part of soft palate. The pharyngeal structure consists of the following parts: nasopharynx from the end of the hard palate posterior nasal spine to the posterior margin of the soft palate; oropharynx from the margin of the soft palate to the tip of the epiglottis; and hypopharynx from the tip of the epiglottis to the vocal cords. The tongue structure was the biggest one. Whereas it was clearly visible in the central slices, the observer was rather uncertain in marking the lateral tongue parts, which were adjacent to the cheeks. For the same reason, only the central parts of the soft palate were marked.

In total, data from 181 subjects were manually segmented. The human reader required about two hours per dataset. 20 datasets were randomly selected as the test set. Thereafter, we randomly selected 10 more datasets for validation, and the other 151 datasets were used in training.

## Methods

### Analysis of intra-observer variability

**Fig. 2:**
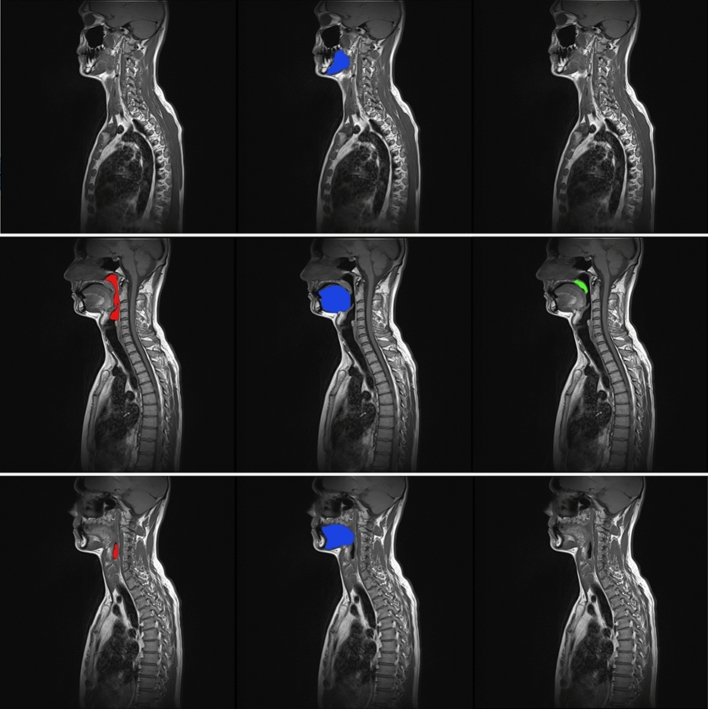
Example slices with overlaid manual segmentation of pharynx (left), tongue (middle), soft palate (right) with original T1-weighted slices 2, 7, 10 of one dataset

In Fig. 2, manual readings for three example slices from one dataset are presented. To evaluate the intra-observer variability, the experienced observer repeated manual segmentation of 20 datasets, which were put to the test set. The manual readings were performed in Horos Software, a free and open source code software (FOSS) program that is distributed free of charge under the LGPL license [23]. The second round of reading was performed in one month after the first one, and the expert could not check the first reading again.

### Automated segmentation in a cascaded framework

**Fig. 3:**
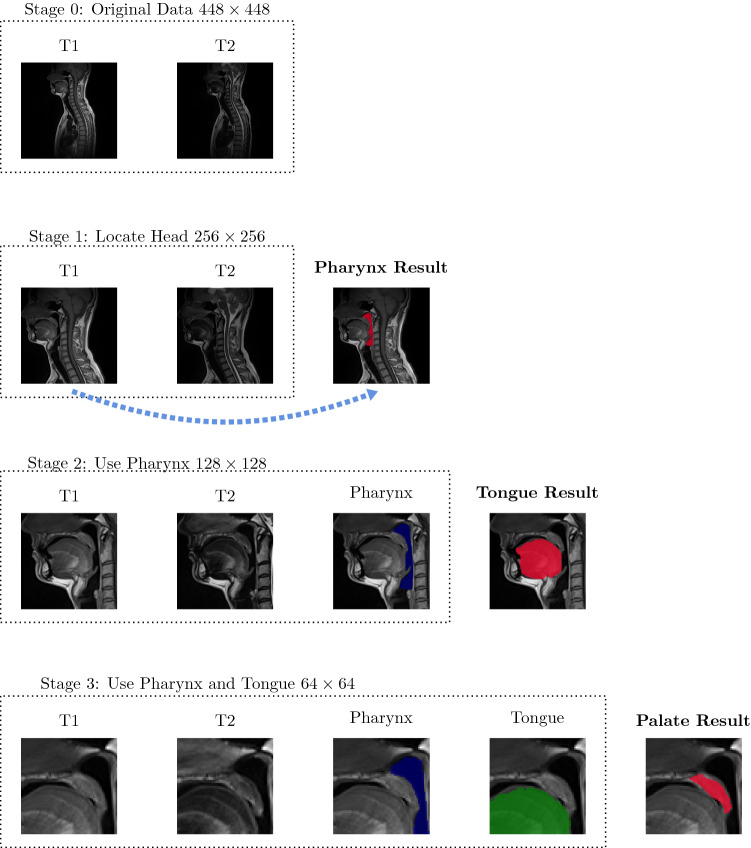
The schematic presentation of the proposed cascade framework for segmentation of pharynx, tongue, and soft palate

As it can be observed in Fig. 1, the slices cover approximately half of a subject’s body, whereas the structures of interest are relatively small. Therefore, we designed and applied a cascaded framework, which allowed for localization of the oral cavity region and efficient segmentation of pharynx, tongue, and soft palate. The cascade stages are schematically presented in Fig. 3. Each stage is described in detail below.

*Stage 0* starts from original data, which cover approximately the upper half of the human body. Since the organs of interest lied within and adjacent to the oral cavity, we applied a procedure to remove the background and the chest. Each slice was cropped to the resolution of $256\times 256$. The cropping procedure was designed as follows. First, a three-dimensional (3D) region with voxel intensity values $\ge$ than a certain threshold $l=50$ was selected. Second, the axis-aligned bounding box (AABB) was constructed around the thresholded result, which allowed for exclusion of the background regions. The left AABB’s boundary line ($x_0$) corresponded to the tip of the nose. The top AABB’s boundary line ($y_0$) corresponded either to the slice top, or the highest point of the head. Finally, 255 pixels were added to $x_0$ and $y_0$, and the right and bottom cropping lines, namely $x_1=x0+255$, $y_1=y_1+255$, were obtained. Of course, it was also checked that $x_1$ and $y_1$ did not exceed the slice dimensions. Otherwise, the start positions $x_0$ and $y_0$ were updated accordingly. Such cropping resulted in slightly different images among subjects and served as basic data augmentation. Additionally, we applied further augmentation methods as rotation and elastic deformation [27]. Finally, each dataset was z-score normalized [17].

*Stage 1* was designed for segmentation of the pharyngeal structure on the head-cropped $256\times 256$ slices. Due to the high slice thickness and limited amount of ground truth data, a 3D segmentation problem was approximated by an encoder–decoder network with 2D convolutions, and the datasets were considered in a slice-wise manner. We ran a series of experiments with several well-known architectures for semantic segmentation. Namely, we applied UNet [27] with different encoders. The general feature of the network is that it consists of the encoding (contracting) and decoding (upsampling) parts. The skip connections are used from the encoder to decoder at each block. We experimented with the number of blocks, which defined the network’s depth. Moreover, VGG, ResNet, and MobileNet architectures for the encoder were tested [4, 28, 33]. The best performance for pharynx segmentation was achieved by using 4 blocks. We observed that the encoders resulted in similar performance and selected a VGG-like structure, which had less parameters, when compared to ResNet18, ResNet34, VGG11, VGG16, VGG19 with BatchNorm (9 to 20M parameters), and with a slightly better performance, when compared to MobileNetV2 encoder (2M parameters only). Our final model had 7M parameters. Each block in the encoder had two convolutional layers with batch normalization and ReLU activation function, and one max pooling layer. In the decoder, the up-convolutional layers were employed. The first layer started with 32 filters, and in the bottleneck part there were 512 filters. We trained several networks with T1 and T2 only inputs and combined the results on the postprocessing step, as well as the combined T1, T2 multi-channel input. Output was a single channel probability map, which was converted to a binary segmentation by thresholding. It was shown that the best performance had been achieved with only T1-weighted sequence on input for the air-filled structure.

*Stage 2* used the detected pharynx as a physiological landmark, which defined the tongue location. Therefore, the input image was cropped to the size of $128 \times 128$. The cropping procedure was designed as follows. First, the axis-aligned bounding box was built around the pharynx mask, which was computed in Stage 1. The box consisted of top, bottom, left, and right boundaries, namely $py_0$, $py_1$, $px_0$, $px_1$. Second, it was taken into account that the possible tongue dimensions could not exceed top, bottom, and right box boundaries, namely $py_0$, $py_1$, and $px_1$, due to the subject’s position and physiology. The position of the tongue tip could not exceed the position of the nose tip $x_0$, which was detected in Stage 0. Therefore, the cropping could be performed. If, for instance, the difference $py_1-py_0$ was smaller than 128, some pixels were added from above and below, such that the cropped region stayed within the original slice dimensions.

Similarly, as the Stage 1, we experimented with different network architectures, network depth, and initializations. The best performing model for tongue segmentation used a VGG-like encoder, T1- and T2-weighted sequences on input, started with 32 filters, and required about 7M of parameters.

*Stage 3* was the final one in the proposed framework. The soft palate is a very small structure, which lies approximately between the tongue and the pharynx part. Here, the detected tongue and pharynx were utilized as physiological landmarks for the soft palate location. The main idea was that the central part of the soft palate lied in the region, where both pharynx and tongue were visible. The approximate target region was defined by the pharynx mask from the top and right sides, and the tongue mask from the left and bottom sides, respectively. Therefore, a cropping procedure to the size of $64\times 64$ was designed. The top and right boundaries were obtained from the pharynx mask ($py_0$ and $px_1$), and 64 pixels were added/subtracted from them to obtain the other two boundaries. It was checked that they did not exceed the tongue mask. Apart from the pre-extraction of the region of interest, a smaller network and additional oversampling of the soft palate slices in training were introduced to alleviate the problem of the strong class imbalance. The U-Net started with 16 filters and used T1- and T2-weighted sequences.

Postprocessing in Stages 1-3 consisted of extracting of the biggest 3D connected component [12] and thresholding of the output probability map. All voxels with probabilities $<0.5$ were set to 0, and the other ones were set to 1. Finally, the Dice coefficient [12] was computed for each dataset.

## Results and discussion

### Analysis of intra-observer variability

**Fig. 4:**
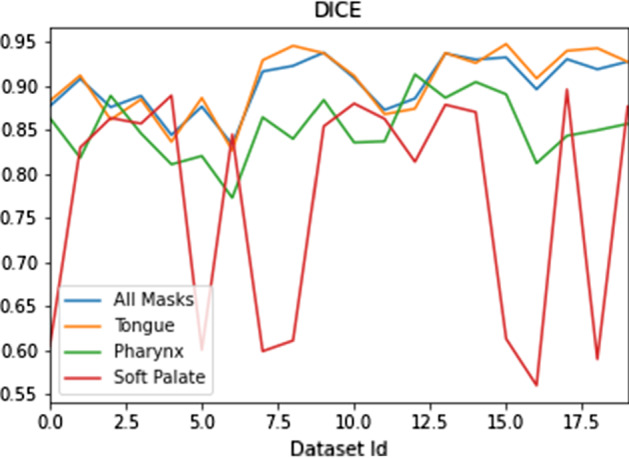
Dice coefficients for 20 datasets measured by the experienced observer in two rounds

In order to fairly evaluate the performance of the automated approach, first we analyzed the human performance. To accomplish this task, our expert executed a double reading of 20 datasets within one-month interval. The datasets were given to the observer in a random order, and during the second reading, it was of course not allowed to view the results of the first one. As it can be observed, the tongue is the biggest segmented structure, and the observer was the most confident in its segmentation. The measurements of the soft palate suffer from the biggest variations due to the small organ’s size. Mean Dice values for 2 measurements are shown in Fig. 4. The averaged Dice values are $0.904 \pm 0.04$, $0.865 \pm 0.035$ , $0.776 \pm 0.13$ for tongue, pharynx, and soft palate, respectively. We aimed that the trained network would reach values lying close to this range.

### Results from the automated framework and discussion

We adapted the implementations provided by [4, 33]. The networks were trained for 100 epochs. Adam optimizer [19] with learning rate of 0.0001, and the Dice loss function [22] were used as default settings. The processing of one dataset with the complete framework and result saving took about 2 seconds using NVIDIA GeForce GTX 1070 with 8Gb.

Since there were two registered modalities available, namely T1- and T2-weighted, it was first investigated, which channel combination would allow for the best performance. We trained three following networks: with T1-weighted as input channel, with T2-weighted as input channel, and the multi-channel input consisting of T1- and T2-weighted images. It is known from the literature that usually the fusion schemes outperform the single-modality schemes, and fusing at the feature level can generally achieve the best performance in terms of both accuracy and computational cost, but can also suffer from the decreased robustness due to the presence of large errors in one or more image modalities [14]. The final (maximal) Dice coefficients for the validation set for pharynx training are 0.875, 0.831, 0.861 for T1-weighted channel, T2-weighted channel, and T1- and T2-weighted channels, respectively. Although our T2-weighted channel did not contain errors, it had more darker regions that lied close to the pharyngeal structures (see Fig. 1), and this obviously confused the network. The combination of two channels performed almost as good as a single T1-weighted input, and the usage of the second channel did not influence much the performance. Therefore, we used our final network with T1-weighted input only. However, we observed that multi-channel input was beneficial for segmentation of soft structures, namely of tongue and soft palate.

We performed the evaluation in several steps. First, each network was trained and evaluated separately, and manual readings were used in Stages 2 and 3 to pre-extract the tongue and soft palate regions. We held out the test set for final analysis and cross-validated the models to assure that the results did not depend on a particular random choice for the train and validation sets. We generated $k=5$ splits of training and validation sets with 10 patients in each validation set and trained *k* models for each stage. The splits were unique, i.e., if a certain patient was used in the validation set for one split, it was used in the training sets for the other splits and did not appear in the other validation sets. The cross-validation results are presented in Table 1. It can be observed that the performance of the models was quite similar for all splits. The largest variation between the splits appears for the soft palate region, which can be explained by the uncertainty of the expert. We took the models from split 0 for further analysis, since its performance was close to the average one of all five models for each organ.

**Table 1:** Dice coefficients for cross-validation of each stage. For each split, 10 patients were selected for validation and the rest was used for training

Mask	Split					$\mu \pm \sigma$
	0	1	2	3	4	
Pharynx	0.892	0.885	0.875	0.903	0.8845	$0.89\pm 0.01$
Tongue	0.91	0.905	0.907	0.899	0.891	$0.9\pm 0.007$
Soft palate	0.84	0.833	0.869	0.825	0.8435	$0.842 \pm 0.016$

**Table 2:** Dice coefficients ($\mu \pm \sigma$) for validation (10 datasets) and test (20 datasets) sets. Left: Each network in the framework was trained and evaluated using the expert ground truth. Right: the cascade framework was executed, and in the intermediate stages the results from the previous stages were employed. Only the final results were compared to the expert ground truth

Mask	$dsc\_\mu \pm dsc\_\sigma$			
	Independent		Cascade	
	Validation set	Test set	Validation set	Test set
Pharynx	$0.89 \pm 0.01$	$0.87 \pm 0.02$	$0.89 \pm 0.03$	$0.87 \pm 0.02$
Tongue	$0.91 \pm 0.03$	$0.89 \pm 0.04$	$0.91 \pm 0.02$	$0.89 \pm 0.03$
Soft palate	$0.84 \pm 0.06$	$0.79 \pm 0.09$	$0.839 \pm 0.05$	$0.79\pm 0.08$

**Fig. 5:**
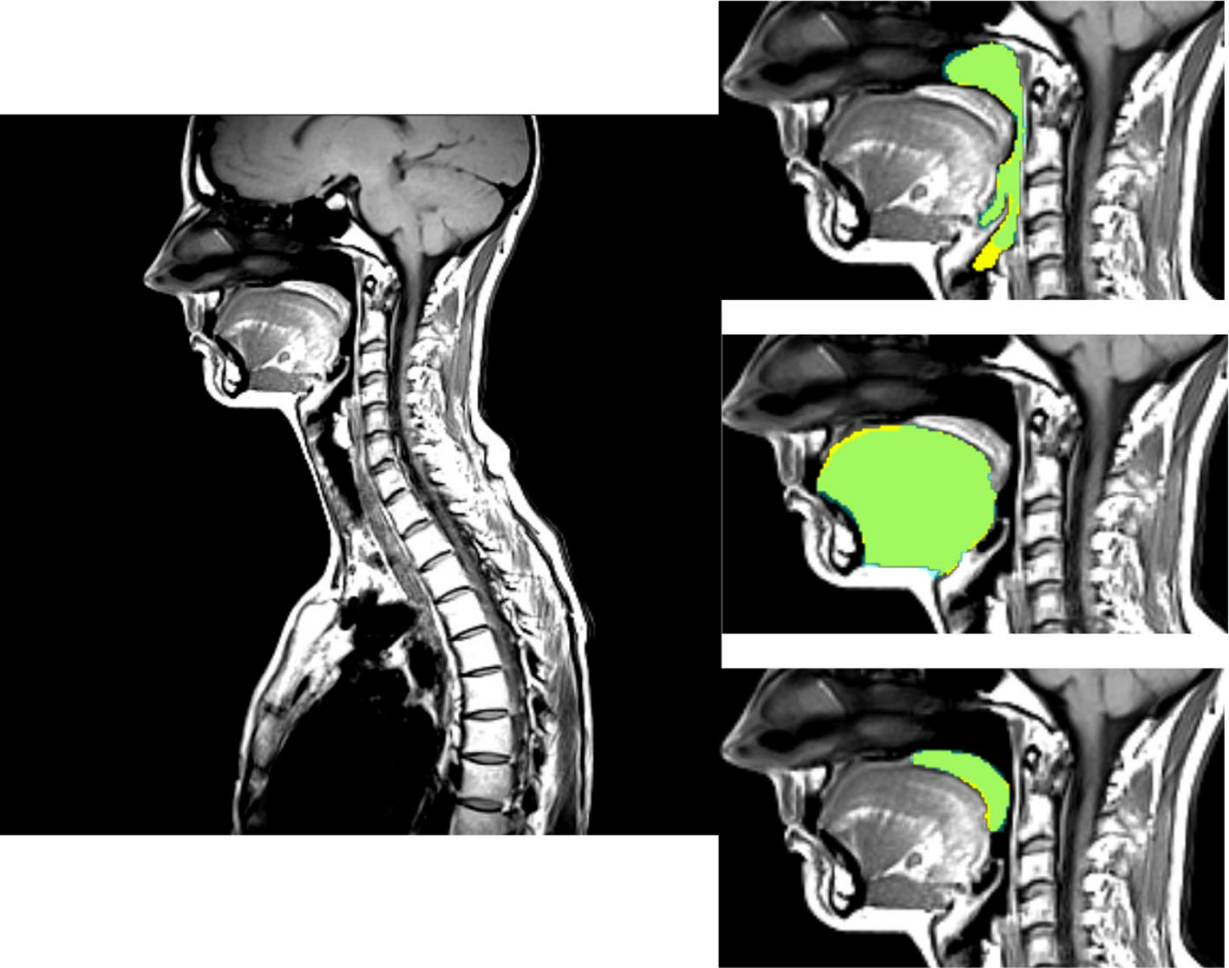
Example T1-weighted slice from a dataset from the validation set. Dice coefficients for pharynx, tongue, and soft palate are 0.9, 0.94, 0.87, respectively. Segmentation results from the automated framework and the human observer are overlaid with the zoomed in regions of interest. True positives (TP) are green; false positives (FP) are yellow, and false negatives (FN) are light blue

Second, we ran the cascade framework in the inference mode to check if the cascade steps might introduce some errors in detection of tongue and soft palate. Namely, the pharynx was detected in the first step, then the computed pharynx mask was used in the second step to localize the tongue, and finally, both pharynx and tongue masks found in the previous steps were used to localize the soft palate location and the soft palate region of interest was fed to the network for segmentation. In Table 2, the Dice coefficients for validation and test set for both setups are given. In Fig. 5, the results from the automated framework and the human reader are overlaid with the T1-weighted slice, as it can be observed that the largest regions are true positives (green), with some minor false positives (shown in yellow) and false negatives (shown in blue).

It can be observed that the cascaded framework using the structures found in Stages 1 and 2 produced results with basically the same accuracy as in the independent setup, where the pharynx and tongue regions are taken from the manual ground truth.

Finally, we compared the results from the proposed framework to the second expert reading provided for 20 datasets. The Dice results were 0.89, 0.87, and 0.756 for tongue, pharynx, and soft palate, respectively. Such values were close enough to the intra-observer variability (Sect. 4.1). These slightly lower values are explained by the fact that the networks were trained with the ground truth provided by the expert in the first measurement round.

Other methods for pharynx segmentation, such as the approaches by Erattakulangara and Lingala [10] and Shahid et al. [30], reported Dice coefficients around $90\%$. Our approach delivered slightly lower Dice coefficients ($87-88\%$), which were fully consistent with the intra-observer difference though. Moreover, these methods were applied to different MR data, therefore, no direct comparison was possible.

**Fig. 6:**
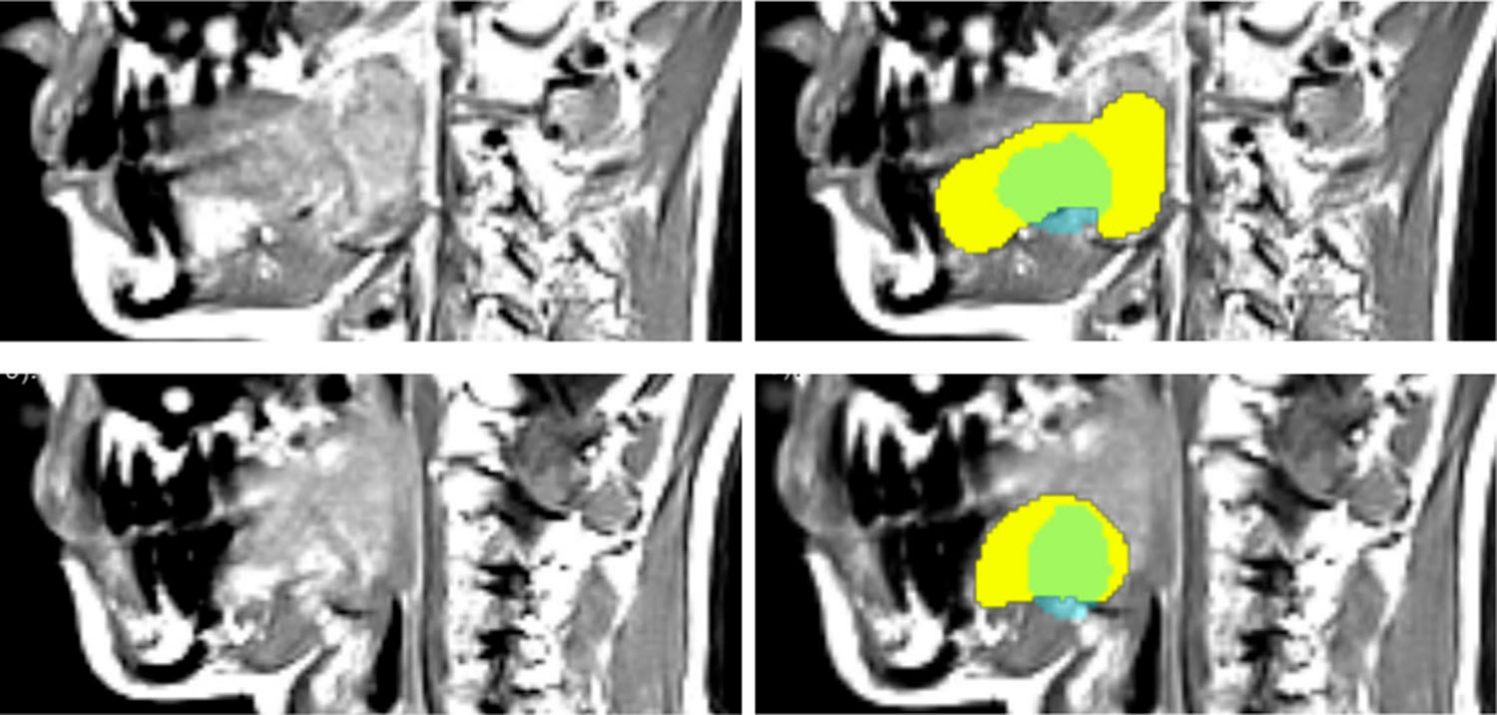
Two close-up views of lateral slices from the T1-weighted dataset from the validation set. Left: the original slice; Right: segmentation results from the automated framework and the human observer are overlaid. True positives (TP) are green; false positives (FP) are yellow, and false negatives (FN) are light blue. The false positive and false negative rates are much higher than in the central slices (cf. Fig. 5)

The tongue segmentation approach [20] was developed for MR images with similar resolution to the data used in our project, and the Dice coefficients were around $90\%$. We observed that the main differences in tongue segmentations appear in lateral slices, where the rest of tongue tissue was not well distinguishable from the other soft tissues in the oral cavity (cf. Fig. 6), and the expert could not deliver the consistent readings there.

**Fig. 7:**
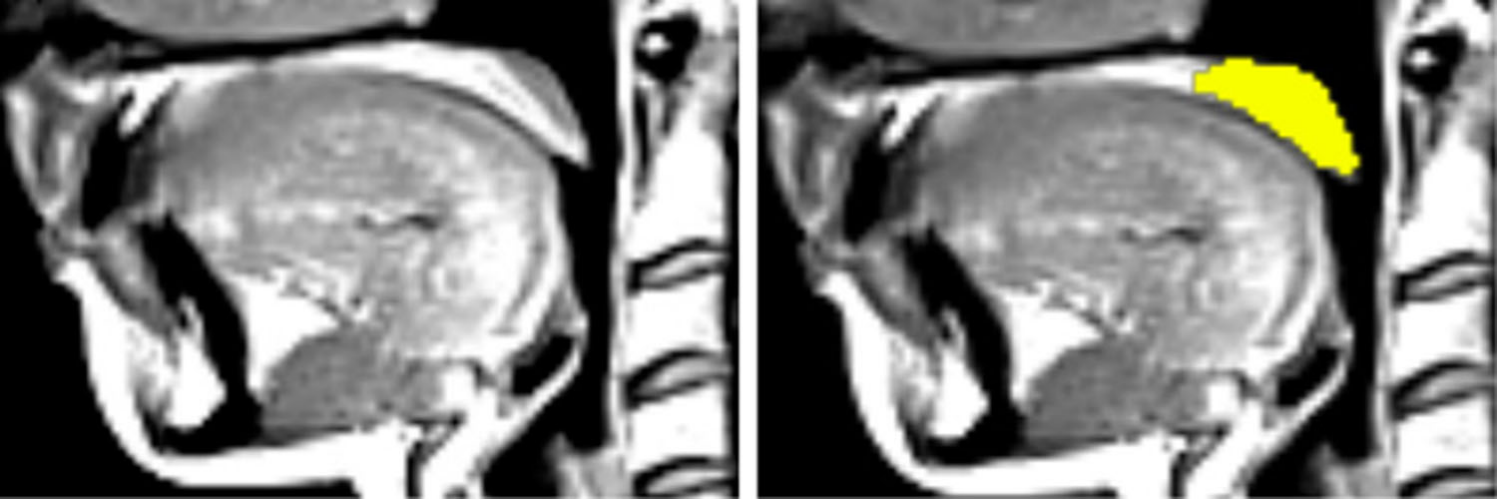
A close-up view of a slice from the T1-weighted dataset from the validation set. Left: the original slice; Right: segmentation results from the automated framework and the human observer are overlaid. True positives (TP) are green; false positives (FP) are yellow, and false negatives (FN) are light blue. Here, the expert omitted this slice, but the framework detected a soft palate region, which was considered as false positives. However, it cannot be claimed that the network detection is wrong, since anatomically it is in the correct position, and the expert had probably rejected the slice due to a relatively low size of the soft palate region

Segmentation of central soft palate parts was rather challenging even for the expert, which was reflected in the intra-observer variability. The main misclassifications appeared due to the fact that this small structure could be delineated only on several slices. However, the expert himself was often unsure, which slices exactly to choose. It is demonstrated in Fig. 4, where the drops in Dice coefficient values for several datasets meant that the expert selected a slice in the first round of soft palate measurement, but missed it in the second round or vice versa. Similar situation was observed for the automated framework. As presented in Fig. 7, the framework detected a soft palate region, but the expert rejected this slice. The network detections, as demonstrated in Fig. 7, were not wrong, though. Moreover, in the other slices, automated results were similar to the manual readings (cf. Fig. 5). Our approach allowed for accurate segmentation, which was comparable to another expert opinion. To our knowledge, the proposed framework is the first one, which considers automatic segmentation of soft palate parts in MR data.

## Conclusions

The deep cascaded framework for efficient segmentation of sleep apnea-relevant structures was presented. The framework consisted of three stages and allowed for segmentation of pharynx, tongue, and soft palate tissues from mid-sagittal T1- and T2-weighted MRI images. The approach produced accurate results, which were consistent with intra-observer variability, and was very fast: namely, the processing of one dataset took around 2 seconds using a modern GPU. The framework will be applied to population-based data available in Study of Health in Pomerania to provide information on the anatomical structures and aspects that constitute important risk factors to the development of OSAS. Such data will be further analyzed to determine the occurrence and severity of OSAS in the general population, as well as aiding in the early detection and management of OSAS patients.
